# Ethnic and gender differences in the management of type 2 diabetes: a cross-sectional study from Norwegian general practice

**DOI:** 10.1186/s12913-019-4557-4

**Published:** 2019-11-28

**Authors:** Anh Thi Tran, Tore Julsrud Berg, Bjørn Gjelsvik, Ibrahimu Mdala, Geir Thue, John Graham Cooper, Kjersti Nøkleby, Tor Claudi, Åsne Bakke, Sverre Sandberg, Anne Karen Jenum

**Affiliations:** 1 Department of General Practice, Institute of Health and Society, University of Oslo, Oslo, Norway; 2 Institute of Clinical Medicine, Faculty of Medicine, University of Oslo, Oslo, Norway; 3 Department of Endocrinology, Morbid Obesity and Preventive Medicine, Oslo University Hospital, Oslo, Norway; 4 Norwegian Quality Improvement of Laboratory Examinations, Haraldsplass Deaconess Hospital, Bergen, Norway; 5 Department of Global Public Health and Primary Care, University of Bergen, Bergen, Norway; 6 Department of Medicine, Stavanger University Hospital, Stavanger, Norway; 7 Department of Medicine, Nordland Hospital, Bodø, Norway; 8 Department of Clinical Biochemistry and Pharmacology, Haukeland University Hospital, Bergen, Norway; 9 General Practice Research Unit (AFE), Department of General Practice, University of Oslo, Institute of Health and Society, Post Box 1130, Blindern, 0318 Oslo, Norway

**Keywords:** Type 2 diabetes, Ethnicity, Gender, Quality of care, General practice and family medicine

## Abstract

**Background:**

Ethnic minority groups from Asia and Africa living in Western countries have a higher prevalence of type 2 diabetes (T2DM) than the general population. We aimed to assess ethnic differences in diabetes care by gender.

**Methods:**

Population-based, cross-sectional study identified 10,161 individuals with T2DM cared for by 282 General Practitioners (GP) in Norway. Ethnicity was based on country of birth. Multilevel regression models adjusted for individual and GP factors were applied to evaluate ethnic differences by gender.

**Results:**

Diabetes was diagnosed at a younger mean age in all other ethnic groups compared with Westerners (men: 45.9–51.6 years vs. 56.4 years, women: 44.9–53.8 years vs. 59.1 years). Among Westerners mean age at diagnosis was 2.7 years higher in women compared with men, while no gender difference in age at diagnosis was found in any minority group. Daily smoking was most common among Eastern European, South Asian and Middle East/North African men. In both genders, we found no ethnic differences in processes of care (GPs’ measurement of HbA1c, blood pressure, LDL-cholesterol, creatinine). The proportion who achieved the HbA1c treatment target was higher in Westerners (men: 62.3%; women: 66.1%), than in ethnic minorities (men 48.2%; women 53.5%). Compared with Western men, the odds ratio (OR) for achieving the target was 0.45 (95% CI 0.27 to 0.73) in Eastern European; 0.67 (0.51 to 0.87) in South Asian and 0.62 (0.43 to 0.88) in Middle Eastern/North African men. Compared with Western women, OR was 0.49 (0.28 to 0.87) in Eastern European and 0.64 (0.47 to 0.86) South Asian women.

Compared with Westerners, the blood pressure target was more often achieved in South Asians and Middle Easterners/North Africans in both genders. Small ethnic differences in achieving the LDL-cholesterol treatment target by gender were found.

**Conclusion:**

Diabetes was diagnosed at a considerably earlier age in both minority men and minority women compared with Westerners. Several minority groups had worse glycaemic control compared with Westerners in both genders, which implies that it is necessary to improve glucose lowering treatment for the minority groups. Smoking cessation advice should particularly be offered to men in most minority groups.

## Background

Ethnic groups originating from Asia and Africa living in Western countries have a higher prevalence of type 2 diabetes (T2DM) than the general population [1] and develop T2DM at a younger age [2–4], increasing the risk of achieving complications relatively early in life [5].

The care of individuals with T2DM and the outcomes are affected by a complex mix of individual factors such as ethnicity, gender, language skills, socioeconomic position, adherence to treatment, health care provider and health care system factors [6, 7]. Ethnic disparities in the care of T2DM have been reported from several countries [4, 8–14]. A large observational study from the Swedish National Diabetes Register showed that minorities of non-Western origin had poorer glycaemic control and a higher risk of developing albuminuria, despite early use of glucose-lowering agents [11]. Another observational study from The Scottish Care Information Diabetes revealed that people with diabetes with Pakistani origin had an increased risk of cardiovascular disease (CVD), whereas those of Chinese origin had a lower risk compared with Caucasians [4]. Similarly, minorities from Asia and the Caribbean living in the UK were less likely to achieve treatment targets for HbA1c, blood pressure (BP) and total cholesterol compared with Caucasians, even after the introduction of financial incentives to improve care [15]. In our study from 2005, we found that the age at the time of diagnosis was 8–15 years younger in Eastern Asians, South Asians and Middle Easterners/North Africans compared with Norwegians [2]. All minority groups had higher mean HbA1c compared with Norwegians [2]. Current diabetes guidelines emphasise that treatment and care should take into account individual needs and preferences [6, 16].

Few studies address ethnic differences in the age at the time of diagnosis and in the management of T2DM by gender [3]. In the present study, we aimed to investigate whether there were ethnic differences in age when diabetes was diagnosed, clinical risk factors, processes of care, prescribed medication and achievement of treatment targets in primary care in Norway. In addition, we analyzed the effect of gender in the different ethnic groups.

## Methods

### Design and setting of the study

We used data from a large population-based, cross-sectional survey, the ROSA 4 study, assessing the quality of diabetes care in general practice in Norway in 2014 [17]. In total, 106 practices with 367 general practitioners (GPs) in three of four health regions in Norway were invited. Of the invited practices, 77 practices with 282 GPs participated the study. Detailed information about the methods is described elsewhere [7, 17]. The ROSA 4 study was approved by the Regional Ethical Committee.

### Participants

In total, 11,428 individuals with a diabetes diagnosis were identified from electronic health records (EHRs). We excluded individuals with other diabetes types than T2DM (*n* = 1183), those from regions with less than 40 individuals (Central and South America, South of Sahara Africa, and Oceania) (*n* = 72), and those with unknown country of birth (*n* = 12), leaving 10,161 individuals with T2DM to be included in the study (See Additional file 1: Figure S1).

### Data collection

Data were collected from January 2015 to April 2016. A software program was used to identify all individuals ≥18 years with a diabetes diagnosis in 2012–2014, and to capture pre-defined data such as results of the blood tests, urine tests and prescriptions of medications from the included GPs’ EHRs. Research nurses examined the EHRs to verify the diabetes diagnosis. In addition they collected clinically relevant data not captured by the electronic software such as year of diagnosis, screening procedures and complications [7, 17].

### Variables

We extracted information from the EHRs about individual level characteristics (age, gender, diabetes duration); processes of care (recorded measurements of HbA1c, BP, LDL-cholesterol, creatinine, albuminuria, body height, body weight, eye examination, foot examination (foot pulse and/or sensation) and smoking habits; medication use (prescriptions of glucose lowering-, antihypertensive- and lipid lowering- agents) and level of HbA1c, BP and LDL-cholesterol. Further, information about coronary heart diseases (CHD) (i.e. angina, myocardial infarction, percutaneous coronary intervention/coronary artery bypass surgery) was extracted and coded as “yes” or “no”. For the majority of variables, the most recently recorded value from October 1st 2013 to December 31st 2014 was used, for eye examination July 1st 2012 to December 31st 2014, and for body height and smoking habits if ever registered. Treatment targets were based on key recommendations in the Norwegian 2009-guildelines: HbA1c ≤53 mmol/mol (7.0%); the intervention threshold for BP was > 140/85 mmHg with treatment target ≤135/80 mmHg while the intervention threshold for LDL-cholesterol was > 3.5 mmol/L with treatment target ≤1.8 mmol/L or 2.5 mmol/L for individuals with or without known coronary heart diseases (CHD) respectively.

In order to investigate ethnic differences in the management of diabetes care, we linked information about country of birth, educational level and number of years resident in Norway, obtained from “Statistics Norway” to the EHR data. Ethnicity was based on country of birth and classified as shown in, Additional file 1: Figure S1) Westerners, 2) Eastern Europeans, 3) Eastern Asians, 4) South Asians, 5) Middle Easterners and North Africans (MENA) and 6) Eastern Africans. Education was categorised as: 1) pre-primary and primary education, 2) secondary education and 3) tertiary education [18].

A questionnaire sent to participating practices provided self-reported information about GP characteristics such as age, gender, specialist status and number of years working as GP in Norway.

### Statistical analyses

We performed analyses stratified by ethnicity and compared differences in clinical characteristics, processes of care, medication use and achievement of treatment targets between ethnic groups, and for all minority groups merged, with Westerners as reference. Further, we performed corresponding ethnic comparisons stratified by gender. Descriptive statistics in the form of frequencies (proportions) and means were used. The Chi-square tests were used to compare ethnic differences in proportions of categorical variables while One-Way ANOVA tests with post hoc tests were used to compare ethnic differences in means of continuous variables with Westerners as reference.

Multilevel regression models were used to account for individuals’ data that were nested within practices. Multilevel binary logistic regression models were fitted to the data on proportions while multilevel linear regression models were fitted to intermediate continuous outcomes. All models were adjusted for individual level characteristics (age, gender, diabetes duration and education), GP level characteristics (gender, specialist status and years working as GP in Norway) and county of residence. We also considered two-way interactions of ethnicity and gender with the outcome measures. However, with the exception of regression models for body height, body weight and smoking habits, the inclusion of these interaction terms did not give better models, hence we did not include the interaction terms. There were no differences in missing data for HbA1c, blood pressure and LDL-cholesterol across ethnicities and gender. Therefore, we included cases with complete data in the regression analyses for mean HbA1c, BP and LDL-cholesterol and achievement of treatment targets. Due to large differences in the mean ages between the Westerners and the other ethnic groups, we also performed a sensitivity analysis for achievement of HbA1c target after having excluded Westerners ≥76 years. When comparing estimates from the minority groups and the reference group, the difference was considered to be significant if the 95% confidence intervals did not overlap. The analyses were performed with SPSS Statistics 24 and StataSE 15.

## Results

### Clinical characteristics

**Fig. 1:**
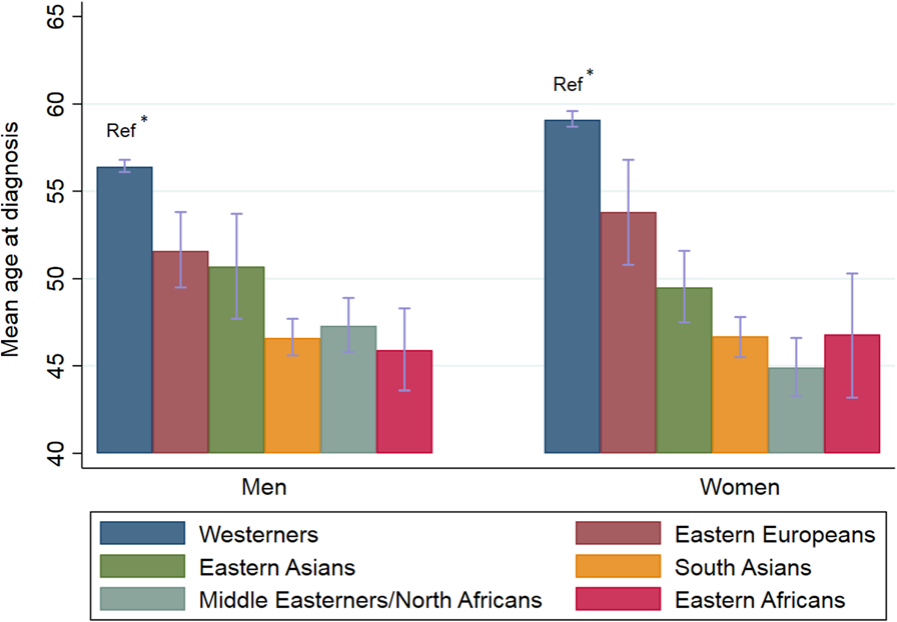
Age at diagnosis by ethnicity and gender. Mean age and 95% CI. The One-Way ANOVA tests with post hoc tests were applied to compare ethnic differences with Westerners as reference. * No overlap in 95% CIs, indicating significant difference between Westerners and the particular minority group

Of the 10,161 individuals with T2DM, 84% were classified as Westerners and 16% as ethnic minorities, with South Asians and Middle Easterners/North Africans as the largest groups. Among the 8492 Westerners, 269 (3.2%) were born outside Norway. All minority groups had significantly lower mean age at the time of diagnosis compared with Westerners (46.4–52.6 years vs. 57.6 years) (Table 1). Among Westerners, the age at diagnosis was lower in men than in women (56.4 years vs. 59.1 years), but no differences in age at diagnosis between men and women were found in the minority groups (Fig. 1).

**Table 1:** Characteristics of individuals with type 2 diabetes by ethnicity and gender

Parameter	Ethnicity					
	Westerners	Eastern Europeans	Eastern Asians	South Asians	MENA ^c^	Eastern Africans
Men, n	4698	103	76	430	200	80
Age (years)	65.1 (64.7 to 65.4)	59.6 (57.4 to 61.9)*	57.8 (54.6 to 60.9)*	56.2 (55.2 to 57.2)*	54.6 (53.0 to 56.2)*	51.7 (49.2 to 54.2)*
Diabetes duration (years)	8.5 (8.3 to 8.7)	8.0 (6.5 to 9.6)	6.9 (5.4 to 8.3)*	9.2 (8.5 to 10.0)	6.6 (5.7 to 7.4)*	6.0 (4.6 to 7.3)*
BMI (kg/m^2^) ^a^	30.1 (29.9 to 30.4)	30.3 (28.9 to 31.8)	26.9 (25.5 to 28.4)*	28.3 (27.7 to 29.0)*	31.2 (29.4 to 33.0)	26.6 (25.0 to 28.1)*
Resident time in Norway (years) ^b^	34.6 (31.9 to 37.3)	22.0 (19.0 to 24.9)*	26.9 (24.7 to 29.2)*	29.5 (28.5 to 30.5)*	21.6 (19.9 to 23.2)*	14.9 (12.8 to 16.9)*
Current smoking (%)	21.9 (20.7 to 23.1)	34.2 (25.0 to 43.4)*	26.6 (16.7 to 36.5)	27.7 (23.5 to 31.9)*	34.8 (28.2 to 41.4)*	21.7 (12.7 to 30.7)
Education (%)						
Pre/primary	28.3 (27.1 to 29.6)	26.0 (17.5 to 34.5)	44.6 (33.4 to 55.8)*	54.8 (50.1 to 59.5)*	46.9 (40.0 to 53.8)*	47.9 (37.0 to 58.8)*
Secondary	51.9 (50.5 to 53.3)	41.7 (32.2 to 51.2)	20.3 (11.3 to 29.3)*	23.2 (19.2 to 27.2)*	28.6 (22.3 to 34.9)*	20.5 (11.7 to 29.3)*
Tertiary	19.8 (18.7 to 20.9)	32.3 (23.3 to 41.3)*	35.1 (24.4 to 45.8)*	22.0 (18.1 to 25.9)	24.5 (18.5 to 30.5)	31.5 (21.3 to 41.7)*
Women, n	3797	81	142	368	140	46
Age (years)	68.3 (67.9 to 68.7)	61.6 (58.8 to 64.4)*	57.7 (55.6 to 59.9)*	56.7 (55.6 to 57.8)*	53.4 (51.6 to 55.2)*	51.9 (49. 3 to 54.9)*
Diabetes duration (years)	9.0 (8.7 to 9.2)	7.3 (6.0 to 8.6)*	7.7 (6.7 to 8.7)*	9.8 (9.0 to 10.6)	7.7 (6.4 to 8.9)	5.5 (4.0 to 6.9)*
BMI (kg/m^2^) ^a^	30.5 (30.2 to 30.8)	31.5 (29.8 to 33.2)	26.3 (25.1 to 27.5)*	31.0 (29.9 to 32.0)	32.2 (30.7 to 33.6)*	30.8 (28.6 to 33.1)
Resident time in Norway (years) ^b^	43.1 (40.3 to 45.9)	22.4 (19.6 to 25.2)*	24.3 (22.7 to 25.9)*	26.5 (25.6 to 27.5)*	21.5 (19.7 to 23.3)*	14.1 (10.9 to 17.3)*
Current smoking (%)	23.9 (22.5 to 25.3)	27.0 (17.3 to 36.7)	12.1 (6.7 to 17.5)*	1.8 (0.4 to 3.2)*	20.2 (13.5 to 26.9)	4.8 (−1.4 to 11.0)*
Education (%)						
Pre/primary	40.2 (38.6 to 41.8)	35.7 (25.3 to 46.1)	49.6 (41.4 to 57.8)	63.4 (58.5 to 68.3)*	73.8 (66.5 to 81.1)*	62.2 (48.2 to 76.2)*
Secondary	45.0 (43.4 to 46.6)	37.1 (26.6 to 47.6)	19.7 (13.2 to 26.2)*	22.5 (18.2 to 26.8)*	14.8 (8.9 to 20.7)*	18.9 (7.6 to 30.2)*
Tertiary	14.8 (13.7 to 15.9)	27.1 (17.4 to 36.8)*	30.7 (23.1 to 38.3)*	14.2 (10.6 to 17.8)	11.5 (6.2 to 16.8)	18.9 (7.6 to 30.2)

Data are mean (95% CI), unless otherwise stated.

^a^ *BMI* Body mass index, ^b^ Resident time for those born outside Norway. ^c^ *MENA* Middle Easterners/North Africans

The chi-square tests and the One-Way ANOVA tests with post hoc tests were applied to compare ethnic differences with Westerners as reference. * No overlap in 95% CIs, indicating significant difference between Westerners and the particular minority group

Ethnic differences in BMI were observed, and women of East Asians had the lowest, while Middle Easterners/North Africans had the highest BMI (Table 1). About 35% of men from Middle East/North Africa and Eastern Europe were daily smokers, compared with 22% in their Western counterparts. Among women, the highest prevalence of daily smokers was found in Eastern Europeans, while very few South Asian and Eastern African women were daily smokers (Table 1).

### Processes of care

Ethnicity had little effect on the GPs performance of the majority of the processes of care after adjustments (Additional file 2: Table S1). Body height and body weight were less often recorded in men in several minority groups, whereas smoking habits were recorded less often in women in most minority groups compared with their Western counterparts. Performance of albuminuria and foot examinations were low in all groups in both genders (Table 2).

**Table 2:** Percentage of individuals with type 2 diabetes receiving specific processes of care by ethnicity and gender

Features recorded in electronic health records % (95% CI)	Ethnicity					
	Westerners	Eastern Europeans	Eastern Asians	South Asians	MENA ^a^	Eastern Africans
Men, n	4698	103	76	430	200	80
HbA1c	89.2 (87.4 to 91.0)	86.5 (78.8 to 94.2)	88.8 (81.5 to 96.1)	92.8 (89.9 to 95.7)	89.2 (84.4 to 94.0)	89.2 (82.1 to 96.4)
Blood pressure	89.0 (87.4 to 90.6)	82.3 (73.9 to 90.8)	90.2 (83.4 to 96.9)	91.6 (88.5 to 94.7)	87.6 (82.8 to 92.6)	84.3 (75.9 to 92.7)
LDL-cholesterol	68.7 (64.7 to 72.8)	71.6 (60.8 to 82.4)	72.8 (60.9 to 84.7)	73.1 (66.7 to 79.4)	73.7 (65.9 to 81.6)	66.4 (53.6 to 79.2)
Creatinine/ e-GFR	82.13 (80.6 to 85.6)	80.0 (71.0 to 89.0)	82.9 (74.4 to 91.8)	84.3 (79.8 to 88.9)	83.7 (78.0 to 89.5)	84.9 (76.9 to 93.0)
Albuminuria	23.4 (17.0 to 29.9)	25.0 (13.5 to 36.5)	25.4 (13.2 to 37.7)	17.6 (11.1 to 24.2)	22.4 (13.5 to 31.2)	25.0 (12.5 to 37.5)
Body height	75.8 (71.1 to 80.6)	64.8 (52.9 to 76.8)	67.8 (55.2 to 80.5)	62.3 (43.0 to 70.3)*	59.5 (48.8 to 68.3)*	51.9 (37.9 to 66.0)*
Body weight	58.1 (52.5 to 63.7)	42.7 (30.3 to 55.2)	48.1 (34.3 to 61.8)	43.9 (35.9 to 51.9)*	40.0 (30.4 to 49.5)*	43.8 (30.0 to 57.5)
Eye examination	60.7 (57.9 to 63.6)	61.8 (51.8 to 71.8)	62.1 (51.6 to 72.6)	62.4 (56.9 to 68.0)	56.4 (48.9 to 63.8)	67.0 (56.4 to 77.6)
Foot examinations	30.3 (26.6 to 34.0)	24.9 (15.0 to 34.9)	22.4 (11.4 to 33.4)	25.6 (19.3 to 31.8)	24.8 (17.1 to 32.4)	24.6 (13.3 to 35.9)
Smoking habits	85.8 (83.0 to 88.7)	83.0 (74.3 to 91.6)	88.0 (79.9 to 96.0)	81.0 (75.2 to 86.7)	85.5 (79.5 to 91.5)	61.4 (47.8 to 75.1)*
Women, n	3797	81	142	368	140	46
HbA1c	91.3 (90.1 to 92.5)	86.2 (77.3 to 95.2)	89.2 (83.2 to 95.2)	91.9 (88.0 to 95.7)	93.7 (88.3 to 99.2)	79.7 (63.7 to 95.5)
Blood pressure	88.7 (87.4 to 90.1)	90.7 (83.6 to 97.9)	89.6 (84.1 to 95.1)	87.1 (82.6 to 91.7)	91.6 (86.2 to 97.1)	85.2 (72.9 to 97.5)
LDL-cholesterol	70.0 (66.3 to 73.8)	59.5 (45.9 to 73.2)	66.7 (56.86 to 76.6)	65.2 (57.6 to 72.8)	62.8 (51.6 to 74.1)	51.7 (32.5 to 70.9)
Creatinine/ e-GFR	87.0 (85.0 to 89.1)	82.7 (73.1 to 92.3)	84.7 (78.1 to 91.3)	86.9 (82.2 to 91.6)	85.0 (77.6 to 92.4)	77.5 (62.5 to 92.4)
Albuminuria	23.6 (17.9 to 29.3)	19.7 (9.2 to 30.2)	23.1 (13.9 to 32.3)	23.7 (16.1 to 31.3)	19.3 (10.6 to 28.0)	19.9 (6.0 to 33.9)
Body height	72.6 (68.1 to 77.1)	76.9 (65.8 to 87.7)	69.7 (60.0 to 79.4)	63.9 (55.9 to 71.9)	66.8 (55.7 to 77.9)	68.3 (49.5 to 87.0)
Body weight	53.1 (47.4 to 58.7)	56.3 (42.7 to 69.9)	43.4 (32.7 to 54.0)	49.0 (40.7 to 57.3)	51.7 (40.2 to 63.2)	41.6 (23.7 to 59.5)
Eye examination	63.1 (64.0 to 66.0)	53.5 (41.5 to 65.4)	73.0 (65.4 to 80.7)	65.0 (59.1 to 70.9)	66.1 (57.5 to 74.8)	69.2 (54.7 to 83.7)
Foot examination	29.3 (25.3 to 33.3)	25.1 (13.0 to 37.1)	25.7 (16.5 to 34.9)	24.7 (17.8 to 31.5)	24.5 (14.5 to 43.5)	25.8 (9.3 to 42.3)
Smoking habits	82.7 (79.4 to 85.9)	83.5 (73.7 to 93.4)	66.5 (65.3 to 76.7)*	55.3 (46.9 to 63.7)*	46.4 (34.9 to 57.8)*	42.5 (23.8 to 61.1)*

^a^ MENA: Middle Easterners/North Africans. Multilevel binary regression models with random effects at general practice level were used to compare the ethnic differences with Westerners as reference, adjusted for individual level characteristics (age, diabetes duration and education), general practitioner level characteristics (gender, specialist status and years working as general practitioner in Norway) and county of residence in Norway. * No overlap in 95% CIs, indicating significant difference between Westerners and the particular minority group

### Medication use

The proportion of minority groups who received prescriptions for glucose-lowering agents and who received three or more glucose-lowering agents was significantly higher than in Westerners (Table 3). However, the proportion of minority groups who received prescriptions for anti-hypertensive was significantly lower than in Westerners. Only Eastern Africans received prescriptions for lipid-lowering agents less frequently than Westerners. When stratified by gender, similar ethnic differences in prescriptions were found (Additional file 3: Table S2).

**Table 3:** Percentage of individuals with type 2 diabetes receiving glucose lowering-, antihypertensive- and lipid lowering medications by ethnicity

Medication % (95% CI)	Ethnicity					
	Westerners	Eastern Europeans	Eastern Asians	South Asians	MENA ^a^	Eastern Africans
N	8495	184	218	798	340	126
Glucose lowering						
Life style modification alone	34.0 (29.0 to 39.0)	25.7 (17.4 to 33.9)	25.2 (17.7 to 32.7)	29.5 (23.4 to 35.7)	27.9 (20.8 to 35.0)	27.5 (17.2 to 37.7)
All agents without insulin	51.5 (49.0 to 54.0)	57.5 (49.8 to 65.2)	62.1 (54.2 to 70.0)*	58.6 (53.8 to 63.4)	63.3 (55.2 to 71.4)*	66.7 (56.6 to 76.7)*
Insulin alone	5.8 (5.2 to 6.4)	6.7 (2.9 to 10.4)	4.3 (1.4 to 7.3)	4.3 (2.8 to 5.8)	2.4 (0.9 to 3.9)*	6.0 (0.8 to 11.1)
Insulin combined with other agents	9.5 (8.6 to 10.3)	11.7 (7.1 to 16.3)	9.9 (5.5 to 14.4)	16.8 (13.8 to 19.7)*	10.4 (7.1 to 13.6)	7.1 (1.9 to 12.4)
Number of agents						
1	35.6 (33.5 to 37.7)	29.2 (20.8 to 37.6)	42.2 (34.0 to 50.4)	36.6 (33.1 to 40.2)	35.1 (27.2 to 43.0)	47.6 (36.1 to 59.2)
2	21.7 (20.4 to 23.0)	26.7 (18.9 to 34.5)	23.6 (18.9 to 28.3)	27.7 (22.9 to 32.5)	23.5 (18.9 to 28.1)	27.4 (17.9 to 36.9)
≥3	9.5 (8.7 to 10.2)	20.1 (12.5 to 27.5)*	10.6 (5.4 to 15.7)	15.2 (12.8 to 17.7)*	17.5 (12.4 to 22.6)*	4.8 (1.2 to 8.3)
Antihypertensive	66.2 (61.5 to 70.8)	60.1 (51.1 to 69.1)	61.7 (53.4 to 69.9)	53.6 (47.1 to 60.1)*	47.4 (39.6 to 55.3)*	36.1 (25.0 to 47.2)*
Lipid lowering	54.1 (49.8 to 58.4)	53.5 (44.3 to 62.7)	47.8 (39.4 to 56.3)	50.6 (44.6 to 56.6)	46.1 (38.7 to 53.5)	24.0 (14.6 to 33.5)*

^a^ MENA: Middle Easterners/North Africans. Multilevel binary regression models with random effects at general practice level were used to compare the ethnic differences with Westerners as reference, adjusted for individuals level characteristics (age, gender, diabetes duration and education), general practitioner level characteristics (gender, specialist status and years working as general practitioner in Norway) and county of residence in Norway. * No overlap in 95% CIs, indicating significant difference between Westerners and the particular minority group

### HbA1c, systolic blood pressure and LDL cholesterol

All minority groups except Eastern Africans had higher mean HbA1c levels, and most minority groups had lower mean systolic BP (SBP) levels and diastolic BP (DBP) levels than Westerners, while no ethnic differences were found for mean LDL-cholesterol (Table 4). Most ethnic differences in HbA1c and blood pressure were also present for both genders (Additional file 4: Table S3).

**Table 4:** Mean HbA1c, blood pressure and LDL-cholesterol with 95% CI in individuals with type 2 diabetes by ethnicity

Variable mean (95%CI)	Ethnicity					
	Western Europeans	Eastern Europeans	Eastern Asians	South Asians	MENA ^a^	Eastern Africans
N	8495	184	218	798	340	126
HbA1c (mmol/mol)	53 (52 to 53)	58 (56 to 61)*	54 (53 to 56)*	56 (55 to 57)*	55 (54 to 57)*	52 (50 to 55)
HbA1c (%)	7.0 (6.9 to 7.0)	7.5 (7.3 to 7.7)*	7.1 (7.0 to 7.3)*	7.3 (7.2 to 7.4)*	7.3 (7.1 to 7.4)*	6.9 (6.7 to 7.2)
Systolic BP (mmHg)	135.9 (135.4 to 136.4)	137.5 (134.6 to 140.3)	132.1 (129.6 to 134.6)*	131.8 (130.4 to 133.2)*	132.9 (130.8 to 135.1)*	132.4 (128.8 to 136.1)
Diastolic BP (mmHg)	78.3 (78.1 to 78.6)	78.3 (76.7 to 79.9)	76.3 (74.9 to 77.7)*	75.6 (74.8 to 76.4)*	75.7 (74.6 to 76.9)*	75.0 (72.9 to 77.0)*
LDL-chol (mmol/L)	2.8 (2.7 to 2.9)	2.9 (2.8 to 3.1)	2.6 (2.5 to 2.8)	2.7 (2.6 to 2.8)	2.7 (2.6 to 2.9)	2.9 (2.7 to 3.1)

^a^ MENA: Middle Easterners/North Africans. Multilevel linear regression models with random effects at general practice level were used to estimate the ethnic differences with Westerners as reference adjusted for individual characteristics (age, gender, diabetes duration and education), general practitioner characteristics (gender, specialist status and years working as general practitioner in Norway) and county of residence in Norway. * No overlap in 95% CIs, indicating significant difference between Westerners and the particular minority group. The number of observations included in univariate/multivariate analyses for mean HbA1c were 9059/8300, respectively. The corresponding number of observations included in regression analyses for mean BP were 8897/8178 and for LDL-cholesterol 6866/6313

### Achievement of treatment targets

**Fig. 2:**
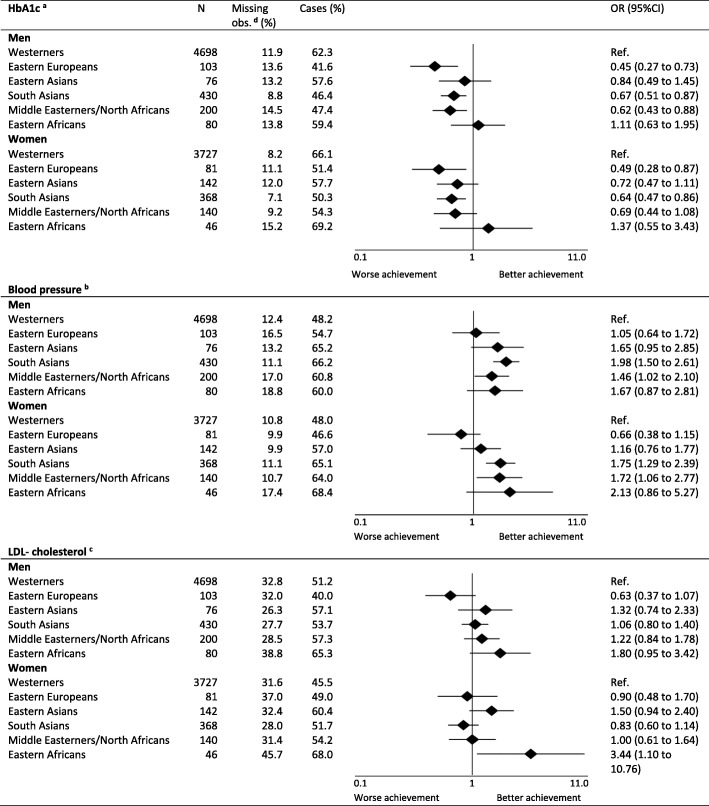
Achievement of treatment targets in individuals with type 2 diabetes by ethnicity and gender. Multilevel binary regression models with random effects at general practice level were used to estimate the difference in the ethnic groups compared to Westerners as reference, adjusted for individual level characteristics (age, diabetes duration and education), general practitioner level characteristics (gender, specialist status and years working as general practitioner in Norway) and county of residence in Norway. ^a^ HbA1c target ≤53 mmol/mol (7.0%). ^b^Blood pressure combined target: ≤ 135/80 mmHg with anti-hypertensives or ≤ 140/85 mmHg without antihypertensive. ^c^ LDL-cholesterol combined target: for individuals with coronary heart disease LDL-chol ≤1.8 mmol/L, without coronary heart disease ≤2.5 mmol/L with lipid lowering medication or ≤ 3.5 mmol/L for individuals without lipid lowering medication. ^d^ Missing observations. The number of observations included in univariate/multivariate analyses for HbA1c target were 9059/8300, respectively. The corresponding number of observations included in regression analyses for blood pressure target were 8897/8178 and for LDL-cholesterol target 6866/6313

The treatment targets were achieved in the following proportions of Westerners: HbA1c 64.0%, blood pressure 48.1% and LDL-cholesterol 48.6%. Compared with Westerners, three minority groups achieved the HbA1c target less often (Eastern Europeans: OR 0.47 (0.33 to 0.69); South Asians: OR 0.65 (0.53 to 0.79); Middle Easterners/North Africans: OR 0.64 (0.48 to 0.84). Similarly, three minority groups achieved the blood pressure target more often than Westerners (South Asians: OR 1.91 (1.56 to 2.35), Middle Easterners/North Africans: OR 1.56 (1.17 to 2.07); Eastern Africans: OR 1.78 (1.09 to 2.89). Only Eastern Africans were more likely to achieve the LDL-cholesterol target compared with Westerners (OR: 2.08 (1.19 to 3.62). Ethnic differences in achievement of the treatment targets by gender are shown in Fig. 2.

## Discussion

Our study is one of few from Europe that provides detailed information about the quality of primary care for T2DM by gender among six different ethnic groups, including groups that have rarely been included in previous studies. The early onset of T2DM in minority groups was also influenced by gender. GPs performance of most of processes of care was comparable between ethnic groups in both genders. Glucose-lowering agents were more often prescribed in minority groups than in Westerners regardless of gender. However, the proportion who reached treatment targets was substantially lower among men born in Eastern Europe, South Asia, Middle East/North Africa and among women born in Eastern Europe and South Asia compared with their Western counterparts. Of particular concern was that daily smoking was more prevalent among Eastern Europeans, and among men born in South Asia and Middle East/North Africa.

T2DM was diagnosed at a considerably younger age in all five minority groups, especially in women, which underscores the increased susceptibility of T2DM in minority women compared to Westerners [19]. This is worrisome as many are in reproductive age when they are diagnosed. Known or undiagnosed diabetes and gestational diabetes, not least in young minority women, may adversely affect pregnancy outcomes for the women and offspring [20]. The ethnic differences in age at onset of T2DM was in accordance with our previous study and studies from other countries [2–4, 11, 21].

Performance of the processes of care is considered to be a quality indicator in diabetes care, as this implies assessment of the risk of developing complications, and may increase the awareness of GPs to intensify treatment when indicated. The state-funded health care service to all citizens implies an equal access to core elements in the public primary health care for T2DM. This may have contributed to the finding of the GPs equal performance for the measurements of HbA1c, BP, LDL-cholesterol, creatinine and albuminuria regardless of ethnicity and gender. The observed ethnic variations by gender in measurements of body height, body weight and the recording of smoking habits may be related to individual factors such as comorbidity, specific needs/wishes for consultations and how the GPs prioritize the time spent in the consultations [22].

A new finding in our study is that Eastern Europeans of both genders emerge as the minority group with the highest HbA1c level after adjustments for education and despite a time of residence in Norway that is comparable to most other minority groups. Importantly, we found smaller differences in mean HbA1c between Westerners and South Asians, Middle Easterners/North Africans in 2014 than in our previous study from 2005 [2] and in most other reports [15, 21] despite the fact that Norwegian GPs are not incentivized to meet treatment targets as in UK and some other countries. More options for intensive treatment in minority groups may have contributed to our observations as more glucose-lowering agents have become available.

Despite the new finding of poor glycaemic control in Eastern Europeans, South Asians still have a higher HbA1c level compared with Westerners, possibly explained by a poorer β-cell function and less ability to compensate adequately for higher glucose levels from insulin resistance [23]. Poor adherence to prescribed blood glucose lowering medication and recommended life style modifications might partly explain why GPs do not manage to achieve treatment targets for glycaemic control [24, 25]. Difficulties in cross-cultural communication between minorities and GPs may also contribute to ethnic differences in self-care management and glycaemic control [26, 27].

Although South Asians and Middle Easterners/North Africans reach BP targets more frequently than Westerners, in agreement with our previous study and reports from Scotland and Sweden [2, 4, 11], GPs should bear in mind that South Asians have a higher risk for CVD at a given BP level compared with Westerners [1].

We found small differences between Westerners and ethnic minorities in mean LDL-cholesterol levels, achievement of LDL-cholesterol targets and the proportion receiving prescriptions for lipid-lowering agents, in accordance with reports from Scotland [4]. Long resident time in Norway among minorities may have enhanced their language skills and contribute to diminish the previously observed ethnic differences in HbA1c level as well as BP and LDL-cholesterol level.

We have also identified ethnic and gender differences in smoking habits which is consistent with the World Health Organization’s report about tobacco use globally [28]. Daily smoking among Eastern Europeans, men born in South Asia and Middle East/North Africa represents an additional risk for cardiovascular complications.

### Strengths and weaknesses

This study has several strengths as it is a large population-based study conducted in general practice with high participation rates for GPs that included all individuals with diabetes on the GP lists. The study population is considered to be representative for the population with T2DM in Norway [7]. Through linkage with data from the governmental based national data source “Statistics Norway”, we have data about ethnicity and could adjust for education. We were also able to collect information about other possible confounders as several GP factors. Not least, the manual verification of diabetes diagnosis and the electronically extracted data by experienced research nurses contributes to the internal validity of this study.

Our study has some limitations as we used cross-sectional EHR data. Some inconsistency between elements of care that is documented (i.e. smoking habits, and weight) and what was actually measured may be present. Although the low number in most minority groups limits the power, particularly for analyses by gender, we found it important also to report these results, as little is known about some of these groups and gender differences. Our results were not adjusted for individual socio-economic status beyond education. We do not have data about how the GPs approached lifestyle management. Further, we lack information about diabetes self-care including lifestyle and compliance to lifestyle modification and prescribed medication. HbA1c is a measure of average glycaemia and is influenced by several factors, i.e. hemoglobin levels and possible ethnic differences in glycation independent of blood glucose levels as previously shown in South Asians [23, 29].

### Implications

For GPs, our findings highlight the importance of timely diagnosis of T2DM among ethnic minorities. Intensive glucose lowering treatment and improved performance of screening procedures for microvascular complications and recording of smoking habits should be prioritized to reduce the risk for future cardiovascular complications. Smoking cessation advice should be frequently offered to most groups of ethnic minority men. Qualitative studies exploring the influences of individuals cultural- and socioeconomic factors on glycaemic control would be of great value to enhance the understanding about differences between the ethnic groups. Future research providing knowledge about the needs of minorities with T2DM in self-care and the GPs challenges in providing optimal diabetes care for minorities in order to develop culturally adapted patient education and tailored education of GPs would be necessary. Further, public health strategies for preventing early onset of T2DM are warranted among ethnic minorities, especially for women.

## Conclusion

We have identified earlier onset of T2DM in all minority groups compared with Westerners, in particular in minority women. We found no gender difference in the age at diagnosis in all minority groups, in contrast to Westerners. The quality of diabetes care in terms of processes of care, was, with few exceptions, equally well performed in all ethnic groups irrespective of gender. Worse achievement of treatment targets for HbA1c in Eastern Europeans, South Asians and men from the Middle East/North Africa and highly prevalent daily smoking among men in several minority groups represents present major concerns.

## Supplementary information

**Additional file 1:** **Figure S1.** Chart of individuals with type 2 diabetes included in the study.

**Additional file 2:** **Table S1.** Performed processes of care for individuals with type 2 diabetes by ethnicity.

**Additional file 3:** **Table S2.** Glucose lowering-, antihypertensive- and lipid lowering medication for individuals with type 2 diabetes by ethnicity and gender.

**Additional file 4:** **Table S3.** Mean HbA1c, blood pressure and LDL-cholesterol with 95% CI in individuals with type 2 diabetes by ethnicity and gender.

## Data Availability

The datasets used and/or analyzed during the current study are available from the corresponding author on reasonable request.

## Abbreviations

BMI: Body mass index
BP: Blood pressure
CHD: Coronary heart disease
CVD: Cardiovascular disease
DBP: Diastolic blood pressure
EHR: Electronic health record
GP: General practitioner
MENA: Middle Easterners/North Africans
MODY: Maturity onset diabetes of the young
SBP: Systolic blood pressure
T2DM: Type 2 diabetes
