# Supplementary material for: Ethnic and gender differences in the management of type 2 diabetes: a cross-sectional study from Norwegian general practice
Source: BMC Health Serv Res. 2019 Nov 28;19:904. doi: 10.1186/s12913-019-4557-4 (PMC6883677; doi:10.1186/s12913-019-4557-4)
Supplement: Supplementary file 1 — Additional file 1: Figure S1. Chart of individuals with type 2 diabetes included in the study. [file 12913_2019_4557_MOESM1_ESM.docx]

**Additional file 1: Figure S1: Flow chart of individuals with type 2 diabetes included in the study**

| Individuals with diabetes in the ROSA 4 study  (n=11 428) | | | |  | |  |
| --- | --- | --- | --- | --- | --- | --- |
|  |  | | |  | Individuals with diabetes other than  type 2 excluded (n=1183):   - Type 1 diabetes (n=1 133) - MODY ^a^ (n=37) - Unknown diabetes type (n=13) |  |
|  |  | |  | |  |  |
|  |  | |  | |  |  |
| Individuals with type 2 diabetes,  born in 103 countries (n=10 245) | | | |  | |  |
|  | |  | |  | Individuals from geographical regions with low numbers with type2 diabetes excluded (n=84):   - Central America (n=7) - South America (n=31) - Africa South of Sahara (n=32) - Oceania (n=2) - Unknown country of birth (n=12) |  |
|  | |  | |  |  |  |
|  | |  | |  |  |  |
|  | |  | |  |  |  |
| Individuals with type 2 diabetes (n=10 161) classified as:   - Westerners (n=8 495) - Eastern Europeans (n=184) - Eastern Asians (n=218) - South Asians (n=798) - Middle Easterners/North Africans (n=340) - Eastern Africans (n=126) | | | |  |  |  |

^a^ MODY: maturity onset diabetes of the young; Westerners: primarily born in Norway, Sweden, Denmark, Finland, Netherlands, the UK, Germany or the US; Eastern Europeans: primarily born in Poland, Bosnia-Hercegovina, Kosovo, Makedonia, Russia or Serbia; Eastern Asians: primarily born in Vietnam, Philippines, Thailand, China or South Korea; South Asians: born in Pakistan, Sri-Lanka, India or Bangladesh; Middle Easterners and North Africans: primarily born in Iraq, Iran, Turkey, Palestine or Morocco; Eastern Africans: born in Somalia, Eritrea or Ethiopia.
