# Supplementary material for: Ethnic and gender differences in the management of type 2 diabetes: a cross-sectional study from Norwegian general practice
Source: BMC Health Serv Res. 2019 Nov 28;19:904. doi: 10.1186/s12913-019-4557-4 (PMC6883677; doi:10.1186/s12913-019-4557-4)
Supplement: Supplementary file 2 — Additional file 2: Table S1. Performed processes of care for individuals with type 2 diabetes by ethnicity. [file 12913_2019_4557_MOESM2_ESM.docx]

**Additional file 2: Table S1. Performed processes of care for individuals with type 2 diabetes by ethnicity**

| Features recorded  in electronic  health records  % (95%CI) | **Ethnicity** | | | | | |
| --- | --- | --- | --- | --- | --- | --- |
|  | Westerners | Eastern Europeans | Eastern Asians | South Asians | MENA ^a^ | Eastern Africans |
| N | 8495 | 184 | 218 | 798 | 340 | 126 |
| HbA1c | 90.0  (88.5 to 91.5) | 86.9  (81.2 to 92.7) | 89.1  (84.4 to 93.8) | 92.5  (90.1 to 94.9) | 90.8  (87.1 to 94.5) | 86.6  (79.7 to 93.5) |
| Blood pressure | 88.8  (87.4 to 90.1) | 86.3  (80.6 to 91.9) | 89.7  (85.3 to 94.1) | 89.5  (86.7 to 92.3) | 89.3  (85.6 to 93.0) | 84.5  (77.5 to 91.5) |
| LDL-cholesterol | 69.3  (65.6 to 73.0) | 66.7  (57.8 to 75.7) | 69.9  (62.0 to 77.8) | 69.3  (63.7 to 74.8) | 69.9  (63.0 to 76.9) | 61.0  (49.9 to 72.1) |
| Creatinine | 84.8  (82.5 to 87.0) | 81.4  (74.7 to 88.1) | 83.7  (78.1 to 89.3) | 85.4  (81.8 to 88.9) | 84.7  (80.1 to 89.4) | 83.3  (76.1 to 90.5) |
| Albuminuria | 22.3  (16.2 to 28.4) | 21.6  (12.8 to 30.3) | 23.0  (14.5 to 31.4) | 19.3  (13.0 to 25.5) | 20.0  (12.8 to 27.2) | 21.6  (11.8 to 31.4) |
| Body height | 74.2  (69.6 to 78.9) | 70.4  (61.5 to 79.3) | 69.4  (61.0 to 77.9) | 62.9  (56.1 to 69.7) | 61.2  (53.0 to 69.4)* | 56.0  (44.2 to 67.9) * |
| Body weight | 55.8  (50.2 to 61.4) | 50.1  (40.0 to 60.1) | 45.4  (36.2 to 54.7) | 46.1  (39.2 to 53.1) | 44.4  (36.1 to 52.6) | 42.7  (31.3 to 54.1) |
| Eye examination | 61.5  (58.8 to 64.1) | 58.7  (50.9 to 66.5) | 68.1  (61.6 to 74.5) | 63.0  (58.6 to 67.4) | 60.4  (54.5 to 66.3) | 68.3  (59.7 to 76.9) |
| Foot examination | 29.9  (26.2 to 33.6) | 25.4  (17.4 to 33.5) | 24.3  (17.0 to 31.6) | 25.2  (20.0 to 30.4) | 24.5  (18.1 to 30.9) | 25.4  (15.7 to 35.1) |
| Smoking habits | 84.3  (81.2 to 87.3) | 83.2  (76.4 to 90.1) | 77.2  (69.9 to 84.5) | 70.0  (63.8 to 76.2)* | 71.5  (64.1 to 78.8)* | 53.1  (41.3 to 65.0)* |

^a^ MENA: Middle Easterners/North Africans. Multilevel binary regression models with random effects at general practice level were used to compare the ethnic differences with Westerners as reference, adjusted for individual level characteristics (age, gender, diabetes duration and education), general practitioner level characteristics (gender, specialist status and years working as general practitioner in Norway) and county of residence in Norway. * No overlap in 95% CIs, indicating significant difference between Westerners and the particular minority group.
