# Supplementary material for: Ethnic and gender differences in the management of type 2 diabetes: a cross-sectional study from Norwegian general practice
Source: BMC Health Serv Res. 2019 Nov 28;19:904. doi: 10.1186/s12913-019-4557-4 (PMC6883677; doi:10.1186/s12913-019-4557-4)
Supplement: Supplementary file 3 — Additional file 3: Table S2. Glucose lowering-, antihypertensive- and lipid lowering medication for individuals with type 2 diabetes by ethnicity and gender. [file 12913_2019_4557_MOESM3_ESM.docx]

**Additional file 3: Table S2. Glucose lowering-, antihypertensive- and lipid lowering medication for individuals with type 2 diabetes by ethnicity and gender**

| **Medication**  **% (95% CI)** | | | | | **Ethnicity** | | | | | |
| --- | --- | --- | --- | --- | --- | --- | --- | --- | --- | --- |
|  |  |  |  |  | Westerners | Eastern Europeans | Eastern Asians | South Asians | MENA ^a^ | Eastern Africans |
| **Men, n** | | | | | 4698 | 103 | 76 | 430 | 200 | 80 |
|  | Glucose lowering | | | |  |  |  |  |  |  |
|  |  | | Life style modification alone | | 31.7  (27.2 to 36.1) | 23.4  (13.2 to 33.5) | 27.4  (16.4 to 38.4) | 28.1  (21.4 to 34.8) | 24.9  (17.2 to 32.5) | 27.1  (15.4 to 38.8) |
|  |  | | All agents  without insulin | | 52.5  (49.7 to 55.4) | 56.6  (47.6 to 65.6) | 56.5  (45.4 to 67.7) | 58.5  (52.0 to 65.0) | 62.6  (56.0 to 69.2)* | 63.6  (51.4 to 75.9) |
|  |  | | Insulin alone | | 5.9  (5.2 to 6.6) | 10.8  (5.2 to 16.5) | 5.8  (0.8 to 10.8) | 3.8  (2.1 to 5.5)* | 1.8  (-0.0 to 3.6)* | 3.0  (-0.6 to 6.6) |
|  |  | | Insulin combined with other agents | | 9.8  (8.8 to 10.8) | 12.0  (6.5 to 17.6) | 10.1  (2.4 to 17.9) | 16.2  (13.0 to 19.4)* | 12.9  (8.2 to 17.6) | 10.6  (2.6 to 18.7) |
|  |  | | Number of agents | |  |  |  |  |  |  |
|  |  | |  | 1 | 35.2  (32.9 to 37.6) | 39.8  (29.3 to 50.3) | 44.9  (32.6 to 57.3) | 34.6  (28.9 to 40.3) | 33.9  (25.7 to 42.2) | 43.9  (32.3 to 55.6) |
|  |  | |  | 2 | 22.6  (21.0 to 24.2) | 21.7  (13.7 to 29.6) | 17.4  (10.7 to 24.1) | 28.8  (23.5 to 34.2) | 23.4  (16.3 to 30.4) | 28.8  (18.6 to 39.0) |
|  |  | |  | ≥3 | 10.4  (9.4 to 11.3) | 18.1  (9.5 to 26.6) | 10.1  (2.6 to 17.7) | 15.1  (12.0 to 18.3) | 19.9  (13.3. to 26.4)* | 4.5  (0.7 to 8.3)* |
|  | Antihypertensive | | | | 66.7  (62.4 to 71.0) | 56.1  (44.7 to 67.4) | 59.9  (47.8 to 72.0) | 54.9  (47.7 to 62.2)* | 46.3  (37.4 to 55.2)* | 43.3  (30.0 to 56.5)* |
|  | Lipid lowering | | | | 56.3  (52.2 to 60.5) | 57.6  (46.2 to 69.0) | 49.6  (37.0 to 62.2) | 58.6  (51.7 to 65.4) | 54.9  (46.3 to 63.5) | 32.4  (19.6 to 45.1)* |
| **Women, n** | | | | | 3797 | 81 | 142 | 368 | 140 | 46 |
|  | Glucose lowering | | | |  |  |  |  |  |  |
|  | |  | Life style modification alone | | 35.1  (30.6 to 39.6) | 28.2  (16.0 to 38.4) | 22.9  (14.4 to 31.3) | 29.2  (22.0 to 36.5) | 29.8  (19.7 to 39.9) | 23.4  (7.0 to 39.8) |
|  | |  | All agents  without insulin | | 49.3  (46.9 to 51.8) | 61.9  (51.4 to 72.4) | 66.7  (57.7 to 75.6)* | 57.5  (53.1 to 61.9)* | 58.3  (45.5 to 71.0) | 66.7  (53.2 to 80.1)* |
|  | |  | Insulin alone | | 5.7  (5.0 to 6.3) | 4.8  (-0.4 to 9.9) | 2.6  (0.3 to 4.9)* | 4.4  (2.2 to 6.6) | 4.9  (1.6 to 8.1) | 13.3  (2.0 to 24.6) |
|  | |  | Insulin combined with other agents | | 9.5  (8.5 to 10.5) | 6.3  (1.2 to 11.5) | 10.3  (6.1 to 14.5) | 17.7  (13.0 to 22.4)* | 11.7  (6.8 to 16.5) | 5.7  (1.8 to 9.5) |
|  | |  | Number of agents | |  |  |  |  |  |  |
|  | |  |  | 1 | 35.4  (33.3 to 37.6) | 20.6  (12.1 to 29.2)* | 42.7  (34.2 to 51.3) | 36.4  (31.8 to 41.0) | 35.0  (22.5 to 47.4) | 46.7  (25.0 to 68.3) |
|  | |  |  | 2 | 21.2  (19.7 to 22.7) | 34.9  (21.7 to 48.1) | 26.5  (19.3 to 33.7) | 29.5  (23.6 to 35.6)* | 26.2  (20.2 to 32.2) | 30.0  (12.5 to 47.5) |
|  | |  |  | ≥3 | 7.9  (6.9 to 8.9) | 17.5  (7.2 to 27.7) | 10.3  (4.6 to 15.9) | 13.6  (11.0 to 16.2)* | 13.6  (7.4 to 19.8) | 3.3  (-3.1 to 9.9) |
|  | Antihypertensive | | | | 67.6  (63.3 to 72.8) | 66.7  (54.9 to 78.5) | 65.0  (55.7 to 74.2) | 55.1  (47.5 to 62.7)* | 51.2  (40.3 to 62.1)* | 24.5  (6.8 to 42.2)* |
|  | Lipid-lowering | | | | 52.8  (49.0 to 56.6) | 49.6  (36.5 to 62.7) | 45.9  (35.9 to 55.9) | 42.9  (35.7 to 50.2) | 35.6  (25.4 to 45.8)* | 13.4  (0.9 to 25.9)* |

^a^ MENA: Middle Easterners/North Africans. Multilevel binary regression models with random effects at general practice level were used to compare the ethnic differences with Westerners as reference, adjusted for individuals level characteristics (age, diabetes duration and education), general practitioner level characteristics (gender, specialist status and years working as general practitioner in Norway) and county of residence in Norway. * No overlap in 95% CIs, indicating significant difference between Westerners and the particular minority group.
