# Supplementary material for: Ethnic and gender differences in the management of type 2 diabetes: a cross-sectional study from Norwegian general practice
Source: BMC Health Serv Res. 2019 Nov 28;19:904. doi: 10.1186/s12913-019-4557-4 (PMC6883677; doi:10.1186/s12913-019-4557-4)
Supplement: Supplementary file 4 — Additional file 4: Table S3. Mean HbA1c, blood pressure and LDL-cholesterol with 95% CI in individuals with type 2 diabetes by ethnicity and gender. [file 12913_2019_4557_MOESM4_ESM.docx]

**Additional file 4: Table S3. Mean HbA1c, blood pressure and LDL-cholesterol with 95% CI in individuals with type 2 diabetes by ethnicity and gender**

| Variable | | **Ethnicity** | | | | | |
| --- | --- | --- | --- | --- | --- | --- | --- |
|  |  | Westerners | Eastern Europeans | Eastern Asians | South Asians | MENA ^a^ | Eastern Africans |
| **Men, n** | | 4698 | 103 | 76 | 430 | 200 | 80 |
|  | HbA1c (mmol/mol) | 53  (53 to 54) | 60  (56 to 63)* | 55  (52 to 58) | 56  (55 to 58)* | 57  (55 to 60)* | 51  (48 to 54) |
|  | HbA1c (%) | 7.0  (7.0 to 7.1) | 7.6  (7.3 to 7.9)* | 7.2  (6.9 to 7.5) | 7.3  (7.2 to 7.5)* | 7.4  (7.2 to 7.6)* | 6.8  (6.5 to 7.1) |
|  | Systolic BP (mmHg) | 135.4  (134.8 to 136.1) | 136.4  (132.6 to 140.2) | 131.7  (127.7 to 135.7) | 131.2  (129.4 to 133.1)* | 132.0  (129.3 to 134.6)* | 132.7  (128.4 to 137.1) |
|  | Diastolic BP (mmHg) | 79.1  (78.7 to 79.5) | 78.3  (76.1 to 80.4) | 76.3  (74.0 to 78.6)* | 76.4  (75.4 to 77.5)* | 76.5  (75.0 to 78.0)* | 75.6  (73.1 to 78.1)* |
|  | LDL-chol (mmol/L) | 2.6  (2.6 to 2.7) | 2.8  (2.6 to 3.0) | 2.6  (2.3 to 2.8) | 2.5  (2.4 to 2.6) | 2.6  (2.5 to 2.8) | 2.8  (2.5 to 3.1) |
| **Women, n** | | 3797 | 81 | 142 | 368 | 140 | 46 |
|  | HbA1c (mmol/mol) | 52  (52 to 53) | 58  (55 to 62)* | 54  (52 to 56) | 56  (54 to 57)* | 55  (52 to 57) | 54  (50 to 58) |
|  | HbA1c (%) | 6.9  (6.9 to 7.0) | 7.5  (7.2 to 7.8)* | 7.1  (6.9 to 7.3) | 7.3  (7.1 to 7.4)* | 7.2  (6.9 to 7.4) | 7.1  (6.7 to 7.5) |
|  | Systolic BP (mmHg) | 136.4  (135.6 to 137.1) | 138.9  (134.5 to 143.2) | 133.1  (129.8 to 136.4) | 132.6  (130.4 to 134.9)* | 134.5  (131.0 to 138.0) | 130.8  (124.2 to 137.4) |
|  | Diastolic BP (mmHg) | 77.4  (77.0 to 77.8) | 78.4  (76.1 to 80.8) | 76.1  (74.3 to 77.9) | 74.7  (73.5 to 75.9)* | 74.9  (73.0 to 76.8)* | 73.9  (70.3 to 77.5) |
|  | LDL-chol (mmol/L) | 2.9  (2.8 to 2.9) | 3.1  (2.8 to 3.4) | 2.7  (2.5 to 2.9) | 2.9  (2.7 to 3.0) | 2.9  (2.6 to 3.1) | 2.9  (2.4 to 3.3) |

^a^ MENA: Middle Easterners/North Africans. Multilevel linear regression models with random effects at general practice level were used to estimate the ethnic differences with Westerners as reference, adjusted for individual characteristics (age, diabetes duration and education), general practitioner characteristics (gender, specialist status and years working as general practitioner in Norway) and county of residence in Norway. * No overlap in 95% CIs, indicating significant difference between Westerners and the particular minority group.
